# Research progress on tumor-infiltrating lymphocyte therapy for cervical cancer

**DOI:** 10.3389/fimmu.2025.1524842

**Published:** 2025-05-19

**Authors:** Wei Zhang, Yong-Min Liu, Dong Li, Shan Liu, Xiao-Jun Cai, Ji-Ying Tang, Zhi-Gang Zuo, Xin-Hui Li, Yi Zhao

**Affiliations:** ^1^ Department of Oncology, The First Affiliated Hospital of Dalian Medical University, Dalian, Liaoning, China; ^2^ Department of Oncology, Renmin Hospital, Hubei University of Medicine, Shiyan, Hubei, China; ^3^ Institute of Clinical Medicine, Renmin Hospital, Hubei University of Medicine, Shiyan, Hubei, China

**Keywords:** cervical cancer, tumor-infiltrating lymphocytes (TILs), immunotherapy, TILs therapy, human papillomavirus

## Abstract

Cervical cancer is a common malignant tumor in women, and human papillomavirus (HPV) infection is a major cause of cervical cancer. Tumor-infiltrating lymphocytes (TILs) are a heterogeneous group of lymphocytes primarily composed of T lymphocytes found within the tumor parenchyma and stroma. These cells can be isolated from tumor tissue, activated, expanded *in vitro*, and reinfused into the patient to exert an anti-tumor immune effect. As a form of personalized immunotherapy, TILs therapy has shown satisfactory efficacy and safety in advanced recurrent and metastatic cervical cancer, offering new hope to patients with advanced cervical cancer. However, TILs therapy for advanced cervical cancer still faces several limitations and challenges. This article reviews the process and latest developments in TILs therapy for advanced cervical cancer and discusses the challenges in the usage and prospects for this treatment.

## 1 Introduction

Cervical cancer is a common malignant tumor in the female reproductive system, ranking first among the three major gynecological cancers and fourth among the causes of cancer-related death in women. In 2020, there were approximately 604,000 new cases and 342,000 deaths worldwide, of which 109,700 new cases and 59,000 deaths were in China (1). The treatment of cervical cancer includes surgery, radiotherapy, chemotherapy, targeted therapy, and immunotherapy. Early-stage cervical cancer can be treated with surgery, whereas treatment of advanced cervical cancer primarily relies on systemic treatments such as chemoradiotherapy, targeted therapy, and immunotherapy.

Based on the results of the GOG240 and JGOG0505 studies, the anti-angiogenic drug bevacizumab has been used in the treatment of advanced recurrent and metastatic cervical cancer, for which it has improved patient survival rates. Nevertheless, the prognosis for advanced cervical cancer remains poor, with a five-year survival rate of only about 17% (2). In recent years, immunotherapy has been applied to various cancers with remarkable efficacy, revolutionizing current cancer treatments. Studies such as Keynote-826 and Checkmate-358 have shown that immune checkpoint inhibitors (ICIs) can effectively improve the prognosis of patients with advanced cervical cancer. Many clinical trials have demonstrated that immunotherapy has achieved promising results in treating cervical cancer.

Adoptive cell therapy (ACT) involves isolating, culturing, and expanding T cells from tissues to obtain a large number of highly effective tumor-reactive T cells that can target tumor antigens and efficiently recognize and eliminate tumor cells (3). Currently, three main types of ACTs are in clinical use: tumor-infiltrating lymphocytes (TILs) therapy, T-cell receptor–engineered T cell (TCR-T) therapy, and chimeric antigen receptor T (CAR-T) cell therapy. In solid tumor treatment studies, TILs therapy is the most widely used therapy, achieving promising results in clinical research despite challenges related to specificity and efficacy. In February 2024, Iovance’s AMTAGVI (lifileucel) received FDA approval for advanced melanoma (4), making it the first approved TILs therapy. In advanced cervical cancer, TILs therapy has shown impressive potential, making it the most promising treatment option. This article reviews its research progress and applications, and provides research direction for the treatment of advanced cervical cancer.

## 2 Human papillomavirus early protein-mediated immune evasion

Human papillomavirus (HPV) is a major cause of cervical intraepithelial neoplasia and cervical cancer, with high-risk types HPV16 and HPV18 being particularly significant. Although 90% of HPV infections regress spontaneously due to immune system activity, HPV can utilize various immune evasion strategies, leading to 5% to 10% of infections progressing to precancerous lesions or cervical cancer, of which 70% are associated with high-risk HPV types (5). The oncogenic effects of HPV are largely mediated by its viral proteins. These early proteins evade immune detection by inhibiting antigen-presenting cells from presenting antigens to T cells, thus suppressing T-cell recognition and activation. Specifically, HPV E5 can retain MHC-I complexes in the Golgi apparatus, thereby preventing their transport to the cell surface, which reduces the presentation of viral peptides to MHC-restricted cytotoxic T lymphocytes, and decreases the recognition by CD8+ T cells (6, 7). E5 can also inhibit the expression of MHC-II molecules, blocking the loading of MHC-II peptides and their transport to the cell surface, thereby reducing the immune recognition ability of infected keratinocytes and affecting antigen delivery to effector T cells (8). Moreover, E5 interacts with calnexin to inhibit the folding and trafficking of CD1d, leading to its proteasomal degradation and reduced surface expression. This suppression of CD1d-restricted invariant natural killer T (iNKT) cell-mediated cytolysis and Th1/Th2 polarization allows HPV-infected cells to evade immune surveillance during early infection (9).

**Figure 1:**
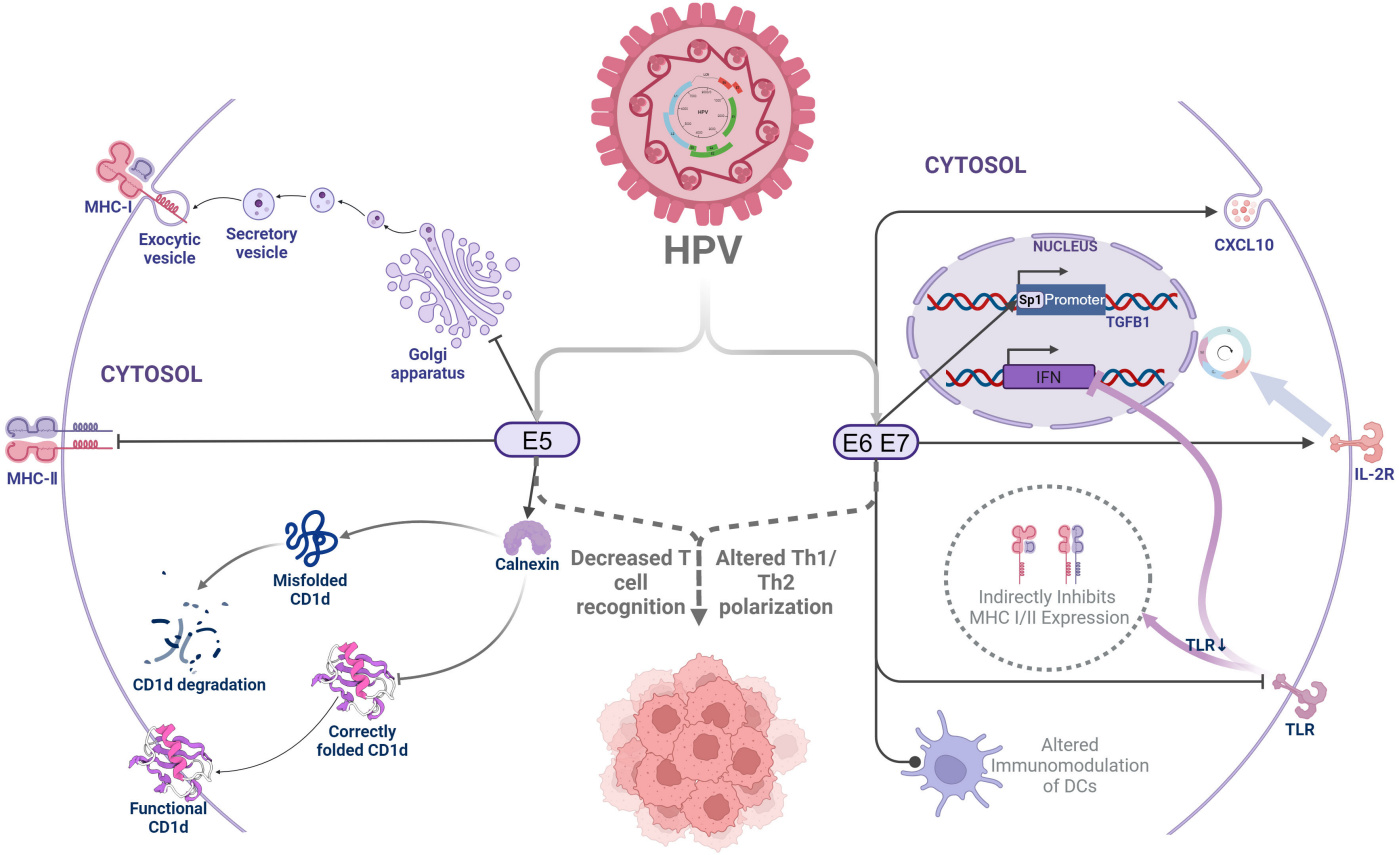
Human papillomavirus early protein-mediated immune evasion. HPV, Human papilloma virus;MHC-I, Major Histocompatibility Complex Class I molecules;MHC-I, Major Histocompatibility Complex Class I molecules;CXCL10, Human Chemokine (C-X-C motif) ligand 10;TLR, Toll-like receptors;IL-2R, Interleukin 2 receptor;IFN, Interferon;TGF-β, Transforming growth factor-β.

High-risk HPV E6 and E7 can also induce cervical cancer cells to secrete chemokine ligand 10 (CXCL 10), activate the JAK-STAT pathway, leading to the upregulation of PD-L1 expression, and produce immune escape by stimulating the interaction between PD-1 and PD-L1 (10). These oncoproteins also downregulate Toll-like receptors, disrupting interferon responses and preventing HPV clearance by the immune system (11). The downregulation of Toll-like receptors indirectly inhibits MHC I/II expression, thereby suppressing T-cell activation. Additionally, high-risk HPV E6 and E7 oncoproteins induce interleukin-2 receptor (IL-2R) expression, which depletes IL-2 to evade immune recognition while promoting self-proliferation (12). Furthermore, HPV E6- and E7-driven epithelial hyperplasia alters epithelial dendritic cells, inhibits T-helper type 1 immunity, and skews T-cell differentiation toward a regulatory or anergic phenotype, further suppressing T-cell function (13) (**Figure 1**).

In contrast, TILs therapy expands specific T cells *in vitro* to high numbers to efficiently target and kill tumor cells. An *in vitro* study identified HPV-specific T cells in 23 out of 54 HPV-positive patients, with immune responses targeting both E6 and E7 proteins (14). Sanja et al. (15) demonstrated that a single intravenous infusion of TILs reactive to HPV E6 and E7 oncoproteins led to durable and complete regression of metastatic cervical cancer. Thus, TILs may mediate tumor regression by directly targeting and recognizing HPV early proteins, thereby exerting an effective anti-tumor immune response.

## 3 Classification of T cells within TILs

The term tumor-infiltrating lymphocytes (TILs) refers to a heterogeneous group of lymphocytes predominantly composed of T lymphocytes found within the tumor parenchyma and stroma. These include T lymphocytes, B lymphocytes, NK cells, dendritic cells (DCs), macrophages and myeloid-derived suppressor cells (MDSCs) (16). The primary components of TILs are tumor-specific T cells, including CD8+ T cells and CD4+ T cells. CD8+ T cells recognize and kill tumor cells, exerting an anti-tumor effect, whereas CD4+ T cells assist in and regulate immune responses but can also inhibit immune reactions, facilitating tumor immune evasion.

### 3.1 CD8+ T cells

As carriers of tumor antigen-specific T-cell receptors (TCRs), cytotoxic CD8+ T cells (CTLs) are the most crucial anti-tumor effector lymphocytes. These TCRs specifically recognize tumor neoantigens presented by MHC-I molecules on the target cell membrane. Upon antigen recognition, CTLs execute their cytotoxic functions by secreting granzyme B and perforin, increasing the synthesis and release of cytokines such as interferon-gamma (IFN-γ) and tumor necrosis factor-alpha (TNF-α) and inducing tumor cell apoptosis via the FasL/Fas pathway (17, 18). IFN-γ, TNF-α, and IL-2 are key cytokines secreted by activated T lymphocytes and play significant roles in TILs therapy. When T cells are cultured *in vitro* during TILs therapy, the proportion of CD8+ T cells targeting tumor antigens is further enriched through continuous artificial tumor antigen presentation.

Among the CD8+ T cell subtypes, Th1 cells, the most typical cytotoxic T cells, are known for secreting high levels of perforin, granzyme B, IFN-γ, and TNF-α and exhibiting potent cytotoxicity (19). Research indicates that Tc1 cells are the predominant subtype found in TILs in patients with malignant melanoma, ovarian cancer, lung cancer, or breast cancer (18). Tc2 cells, which primarily produce type II cytokines, also express high levels of granzyme B and exhibit cytotoxicity comparable to Tc1 cells, making them significant in adoptive transfer anti-tumor therapies (19, 20). In a study of the cytokine expression profiles of CD8+ T cells in the TILs from eight cervical cancer patients, Sheu et al. found that 67% of the CD8+ T cells were Tc2 cells (21). Tc22 cells, another subset within the CD8+ T cell population, secrete granzyme B and possess substantial cytotoxic activity, highlighting their distinct anti-tumor potential in adoptive cell immunotherapy.

### 3.2 CD4+ T cells

CD4+ T cells are a key subset of T lymphocytes that recognize antigens presented by antigen-presenting cells, such as dendritic cells. Specifically binding to MHC-II molecules on the antigen-presenting cells through TCRs, CD4+ T cells primarily play a supportive and regulatory role in immune responses. CD4+ T cells enhance immune responses and anti-tumor effects by secreting cytokines and interacting with other immune cells. CD4+ T cells secrete cytokines such as IFN-γ and TNF, which directly limit tumor growth and modulating the immunogenicity and vascularization of the tumor microenvironment (TME) (22).

Within TILs, CD4+ T cells play crucial roles in supporting and regulating immune responses. Based on their function differentiation, CD4+ T cells can be divided into different subsets, including T helper 1 (Th1), Th2, Th9, Th17, T follicular helper (Tfh), and regulatory T (Treg) cells. Correspondingly, these subsets secrete cytokines and exert distinctive effects on tumor immunity. Th1 cells primarily produce IFN-γ and TNF-α, which activate other immune cells, including CD8+ T cells, macrophages, and B cells (23), and promote cell-mediated immune responses and sustain anti-tumor activity. Th2 cells can either exhibit anti-tumor immune responses or inhibit anti-tumor cell activity, sometimes promoting tumor growth (22). Research suggests that in the immune response to HPV infection, CD4+ T cells tend to favor Th2 responses. The imbalance between Th1 and Th2 cells in HPV-infected individuals might limit immune function, leading to cancer cell proliferation and metastasis. This Th1/Th2 imbalance may be associated with the development and poor prognosis of cervical cancer (24). Th9 cells secrete IL-9, stimulate dendritic cells to uptake and present antigens, and activate CD8+ T cells (23). Th17 cells have high metabolic activity and can transform into Th1 cells. Th17 cells secrete IL-17, IL-22, and other cytokines, activate immune cells and enhance anti-tumor effects (25). Tfh cells mainly promote humoral immunity by facilitating germinal center (GC) responses and differentiation of memory B cells and plasma cells (26). Treg cells exert immunosuppressive effects and play a dual regulatory role in high-risk HPV infections. They form protective physiological defenses against persistent inflammatory stimulation induced by HPV, reducing immune clearance of pathogens and the resulting tissue damage caused by immune imbalance. On the other hand, Treg cells consume IL-2 and release inhibitory cytokines such as TGF-β and IL-10, leading to T-cell apoptosis and promoting tumor growth. Therefore, elevated Treg levels are associated with increased risk of cervical cancer (22, 27).

CD4+ T cells also have immune memory capabilities, enabling them to persist and quickly respond when re-exposure to the same antigen (22). This allows them to maintain long-lasting immune responses against tumors and mount rapid attacks when needed. Although CD4+ T cells were traditionally thought to kill tumors primarily by regulating and coordinating with other immune cells, recent research has found that CD4+ effector T cells can independently eliminate established tumors as effectively as CTLs. It has been shown that only a small number of CD4+ effector T cells (about 1% of tumor-infiltrating immune cells) are located at the tumor invasion edge, where they interact with CD11c+MHC-II+ antigen-presenting immune cells and indirectly eliminate tumors. In contrast, a large number of CTLs infiltrate into the tumor core, directly targeting and killing MHC-I-expressing tumor cells (28).

T cells are the primary cells in TILs, and combining CD4+ T-cell and CD8+ T-cell therapies may lead to a more effective immune response against the target. Clinical efficacy may be related to the CD4+/CD8+ T-cell ratio in the infusion. Research data indicate that CD4+/CD8+ T-cell ratio is associated with clinical response in adoptive immunotherapy targeting B-cell maturation antigen (BCMA) in multiple myeloma (29). However, a group of clinical trials related to HPV-associated cancers suggested that the CD4+/CD8+ T-cell composition did not affect outcomes, as clinical responses were observed in infusion products with different CD4+/CD8+ T-cell ratios (30). The cytokines produced by TILs can also activate and proliferate NK cells, generating an anti-tumor immune response. During the preparation of TILs therapy, identifying the cell subsets with the most potent anti-tumor activity can be prioritized for further optimization in cell production, which may help enhance and sustain comprehensive immune response, thereby harnessing the full potential of anti-tumor immunity.

### 3.3 Immune microenvironment of cervical cancer

The tumor microenvironment (TME) refers to a heterogeneous and continuously dynamic assembly of immune cells, stromal cells, vasculature, and extracellular matrix (ECM) that collectively shape the local microenvironment influencing tumor cell proliferation, invasion, and metastasis (31). In cervical cancer, the immune microenvironment exhibits marked heterogeneity across patients, with its composition and functional states being closely linked to pathological subtypes, molecular profiles, and therapeutic responses. Significant differences exist between the immune microenvironments of cervical squamous cell carcinoma (CSCC) and cervical adenocarcinoma (CAde): CSCC exhibits higher infiltration of cytotoxic CD8+ T cells, natural killer (NK) cells, and pro-inflammatory macrophages (M1 phenotype), whereas CAde is dominated by regulatory T cells (Tregs), immunosuppressive macrophages (M2 phenotype), and cancer-associated fibroblasts (CAFs) (32–34). Single-cell RNA sequencing (scRNA-seq) studies further reveal that in CSCC, T cells undergo a phenotypic shift from cytotoxic to exhausted states, whereas CAFs in CAde drive immunosuppression through inflammatory regulation (34, 35). Additionally, germinal center B cells within tertiary lymphoid structures are associated with improved prognosis (36). scRNA-seq analyses demonstrate that CSCC is enriched with cytotoxic CD8+ T cells, effector memory CD8+ T cells, and pro-inflammatory NK cells, whereas CAde is predominantly infiltrated by naïve T cells and immunosuppressive Tregs (34). CAFs in CAde participate in immune modulation through the secretion of inflammatory cytokines, whereas CAFs in CSCC are linked to tumor epithelial-mesenchymal transition (EMT) (34, 35). Furthermore, therapeutic interventions differentially remodel the immune microenvironment of cervical cancer. For instance, concurrent chemoradiotherapy (CCRT) may transiently enhance local CD8+ T cell infiltration, while systemic therapies can induce systemic expansion of immunosuppressive monocytic myeloid-derived suppressor cells (Mo-MDSCs) and Tregs in peripheral blood (37, 38).

## 4 TILs therapeutic workflow

Tumor-infiltrating lymphocytes (TILs) are immune lymphocytes isolated from tumor tissues, originating from the patient’s own immune system. These cells infiltrate malignant tumors, encounter tumor antigens, and mediate cytotoxic responses against tumor cells through tumor antigen-specific TCR recognition, representing a host-specific immune reaction to neoplastic cells (39). TILs therapy involves isolating, extracting, culturing, and expanding autologous immune cells from the patient’s tumor tissue, followed by reinfusion into the patient, with the core principle of harnessing the patient’s own immune cells to combat cancer (40). Prior to TILs reinfusion, patients undergo preparatory regimens, including non-myeloablative (NMA) lymphodepletion and interleukin-2 (IL-2) administration, to prime the tumor microenvironment for optimal antitumor activity of reinfused TILs.

### 4.1 Preparation of TILs

**Figure 2:**
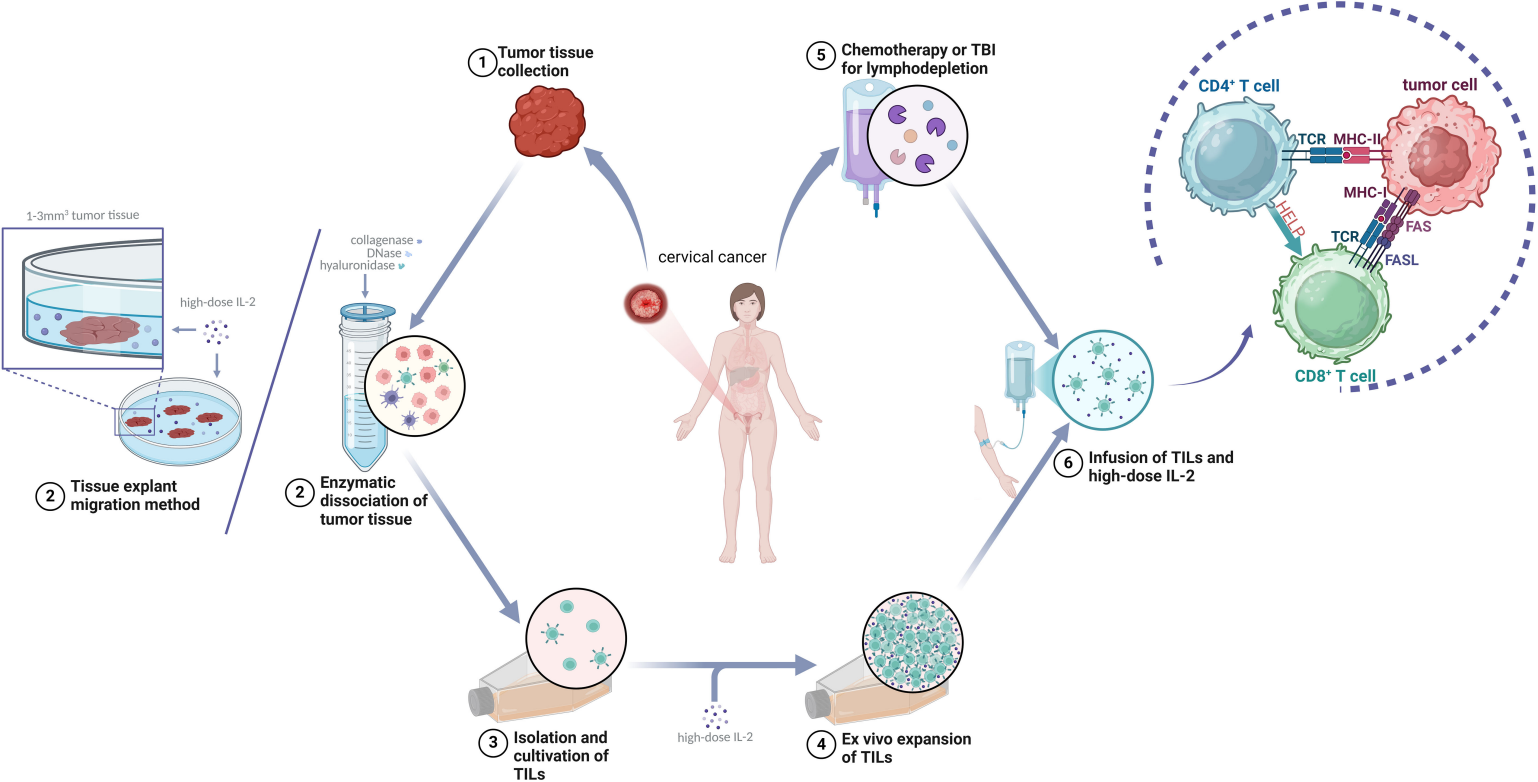
General scheme of tumor-infiltrating lymphocyte (TILs) therapy.

The typical process of TILs preparation includes the following steps: (1) Obtaining tumor tissue: Fresh tumor specimens are obtained via surgical resection or biopsy, followed by meticulous dissection to remove necrotic debris and blood clots under aseptic conditions. This procedure demands meticulous precision and technical expertise to ensure procurement of sufficient viable tumor specimens while minimizing trauma to the patient. (2) Performing the pre-rapid expansion protocol (pre-REP): Following surgical resection, tumor specimens are promptly transferred to the laboratory for processing. The enzymatic digestion protocol involves sequential treatment of tumor fragments with a cocktail of collagenase, DNase, and hyaluronidase to achieve tissue disaggregation. Tumor-infiltrating lymphocytes (TILs) are subsequently isolated through advanced cell sorting methodologies, including flow cytometry-based sorting (e.g., CD3^+^CD8^+^ surface marker selection) or magnetic bead-based isolation (41). Alternatively, the tissue explant migration method entails aseptically mincing tumor specimens into fragments (1–3 mm³) that are evenly distributed in culture dishes. These fragments are maintained in IL-2-supplemented medium (1000–6000 IU/mL) under standardized conditions (37°C, 5% CO_2_) for 1–3 weeks, allowing spontaneous migration of lymphocytes from the tumor periphery into the culture medium, followed by collection of migratory TIL populations (42). The isolation process must be conducted under sterile conditions to ensure TILs purity and activity. (3) Performing the rapid expansion protocol (REP): The isolated TILs are cultured in a medium containing a high dose of IL-2, in which they expand efficiently (approximately 95,652-fold) while retaining strong anti-tumor cytotoxic function (43). Several weeks are required in this process, during which culture conditions, such as temperature, gas composition, and nutrients in the medium, are fine-tuned to optimize TILs growth and activity. The expanded TILs undergo rigorous quality-control tests to ensure their safety and efficacy. Only cells that pass these tests can be used in the subsequent treatment process (**Figure 2**).

Comparative analyses demonstrate that the tissue explant migration method induces significantly less cellular stress than enzymatic digestion protocols, better preserving TIL viability and functional integrity. This approach exhibits particular suitability for cervical carcinoma specimens characterized by low-fibrosis soft tissue architecture. However, its clinical implementation requires optimization of tumor fragment dimensions (typically 1–3 mm³) to maximize lymphocyte migration efficiency, with extended ex vivo expansion timelines (3–4 weeks) representing a notable limitation. To reconcile processing efficiency with cellular fitness, hybrid protocols incorporating partial enzymatic pretreatment followed by explant culture of residual tissue fragments have shown promise in preliminary studies.

After surgical excision, the tumor tissue is surgically removed before TILs are isolated from the excised tumor tissue, selected, and cultured. Using the rapid expansion protocol (REP), the TILs are expanded *in vitro* in the presence of high-dose interleukin-2 (IL-2). The patient undergoes lymphodepletion and receives a high dose of IL-2 before reinfusion of the TILs. After the TILs enter the patient’s body, CD4+ T cells bind to major histocompatibility complex (MHC) II, assisting and regulating the immune response. Cytotoxic CD8+ T cells (CTLs) bind to MHC I and secrete cytokines such as granzyme B, perforin, IFN-γ, and TNF-α as well as mediate tumor cell apoptosis through FasL/Fas interaction.

### 4.2 Pretreatment of TILs therapy

Before TILs infusion, patients undergo an NMA lymphodepletion regimen, commonly referred to as “lymphodepletion,” through chemotherapy or total body irradiation (TBI). The main purpose of lymphodepletion is to eliminate the endogenous lymphocytes, which can significantly enhance the sustained activity of the adoptively transferred T cells. Lymphodepletion also enhances the effect of TILs through two other potential mechanisms. One mechanism is by increasing the number of host homeostatic cytokines, including IL-7 and IL-15, while decreasing the number of endogenous lymphocytes that compete for these trophic cytokines (44, 45). The second mechanism is by enhancing the activation of antigen-presenting cells (APCs), which play a crucial role in regulating adoptively transferred T cells (46). In a clinical study in which infused TILs were labeled with radionuclides and tracked *in vivo* by imaging, patients who had undergone cyclophosphamide lymphodepletion had significantly more TILs migration to the tumor than those who had not undergone this pretreatment (47). Currently, cyclophosphamide + fludarabine (FC) chemotherapy is widely used for lymphodepletion. A recent study comparing different lymphodepletion regimens found that cyclophosphamide (60 mg/m^2^) combined with fludarabine (125 mg/m^2^) provided the best lymphodepletion effect and bone marrow recovery with minimal side effects (48). After lymphodepletion, patients receive the cultured TILs along with a high dose of IL-2.

IL-2 is an important immunomodulatory cytokine that stimulates the growth and survival of effector T cells (49), thereby promoting the growth and activity of TILs. Rosenberg et al. discovered that lymphocytes grown in the presence of IL-2 could lyse fresh syngeneic or autologous tumor cells without affecting normal cells. Moreover, *in vitro* experiments have shown the antitumor activity of TILs is greatly enhanced when cultured in a medium supplemented with high-dose IL-2 (50). Although there is no standardized dose for IL-2, most trials use high-dose IL-2 (600,000 IU/kg or 720,000 IU/kg) administered every eight hours until the patient can no longer tolerate the dose (51, 52). The toxicity of IL-2 is dose-dependent and includes high fever, capillary leak syndrome, and other adverse effects. To reduce the toxicity of high-dose IL-2 without compromising T-cell activity, researchers have explored low-dose IL-2 regimens. Clinical trials have reported sizable tumor regression (30%–63%) in 5 out of 25 patients with 10 different cancer diagnoses treated with TILs and low-dose IL-2 (200,000 IU/kg for 14 days), with no severe IL-2-related adverse effects reported (53). In a Danish pilot trial (54) of six patients with metastatic melanoma treated with TILs, low-dose IL-2 (200,000 IU/kg for 14 days) considerably decreased the treatment-related toxicity, with no grade 3 to 4 IL-2-related AEs reported. Objective clinical responses were observed in two of six treated patients, who had ongoing complete responses lasting over 30 months and over 10 months, and two other patients had stable disease lasting four months and five months. These results suggest that low-dose IL-2 administration in TILs therapy can induce an immune response while significantly reducing toxicity.

However, such promising results were not obtained in a phase II clinical trial (55) in which 12 patients with unresectable metastatic melanoma received TILs therapy with an even lower IL-2 dose (125,000 IU/kg × 12 days). Although no unexpected AEs were observed, only three patients experienced partial clinical responses, and none achieved complete remission. These results suggest that low-dose IL-2 might be associated with lower T-cell activity. When the findings of these previous studies are considered together, they indicate that there is currently not sufficient evidence to conclude that low-dose IL-2 regimens can produce the same sustained response as high-dose IL-2 regimens. Large-scale clinical trials are now needed to identify the optimal dosing regimen.

TILs infusion must be performed under strictly sterile conditions to ensure cell purity and activity. In most clinical trials, TILs therapy is administered intravenously, although some studies have reported other routes, such as intrapleural, intraperitoneal, or intratumoral administration (56, 57). Once infused back into the patient’s body, these specially cultured and expanded immune cells begin to seek out and attack cancer cells. Ideally, TILs effectively recognize and kill tumor cells to achieve the treatment goal.

## 5 Application of TILs in cervical cancer

### 5.1 Historical evolution and clinical development of TILs therapy in cervical cancer

The application of TILs in cancer therapeutics traces back to the 1980s. In 1982, Steven Rosenberg (58), widely considered as the father of ACT, first isolated TILs from a murine tumor model and expanded them *in vitro* in the presence of IL-2. He then reinfused these cells into a tumor-bearing host, leading to a significant anti-tumor response. This study laid the foundation for TILs therapy in treating advanced tumors. In 1986, Rosenberg and his team (50) found that with the combination of cyclophosphamide, TILs, and IL-2, 50% to 100% of mice bearing colon adenocarcinoma were cured of advanced hepatic or pulmonary metastasis. Based on their findings, they concluded that infiltrating lymphocytes are composed of tumor antigen-specific immune cells capable of sustained tumor cell killing. In 1994, a clinical trial of TILs therapy for metastatic melanoma showed promising results, with 10% to 30% of patients achieving complete remission and an overall objective response rate of up to 50% (59).

In 2015, Rosenberg et al. (60) reported the first clinical trial of TILs therapy for cervical cancer. This trial with TILs of nine patients diagnosed with advanced recurrent or metastatic cervical cancer resulted in complete regression in two patients and partial remission in one patient. Remarkably, the complete responses persisted for 22 and 15 months respectively, These groundbreaking results marked the first positive outcome of TILs therapy in cervical cancer, offering a potential alternative treatment for patients with advanced disease who had previously undergone chemotherapy or chemoradiotherapy. Nevertheless, the objective response rate (ORR) in the TILs treatment protocol established by Rosenberg’s laboratory was approximately 33.3%, indicating the need for further optimization and improvement in TILs therapy for cervical cancer.

In 2017, Rosenberg et al. (61) published the results from a phase II single-arm clinical trial (NCT01585428) evaluating TILs therapy in patients with metastatic HPV-associated carcinomas. Among eighteen HPV-positive cervical cancer patients, 27.8% (5/18) achieved objective tumor responses, including two complete remission. Follow-up reports revealed that the two patients who had achieved complete remission survived for over five years. In 2019, another clinical trial of TILs therapy for HPV-associated carcinomas was published involving 29 patients, including 18 with cervical cancer. Of these, two had achieved complete remission and three partial remission, with an ORR of 28% (15).

At the 2019 American Society of Clinical Oncology (ASCO) Annual Meeting, Iovance presented compelling clinical data from the C-145–04 study. Among 27 patients with recurrent, metastatic, or persistent cervical cancer, a single infusion of TILs resulted in an ORR of 44% and a complete response rate of 11%. The disease control rate (DCR) was 85%,with the primary adverse events (AEs) being chills, anemia, and diarrhea (62) and no serious adverse effects were reported, In a Chinese translational investigation of adjuvant TILs following concurrent chemoradiotherapy in patients with cervical cancer (NCT 4443296), TILs from 20 of the 27 patients were successfully expanded, with a feasibility of 74.1%. Of the 12 patients who received TILs following concurrent chemoradiotherapy (CCRT), 9 (75.0%) attained complete responses, with a disease control duration of 9 to 22 months (63). Another report (NCT 04766320) detailed the use of TILs in combination with PD-1 antibody therapy in a patient with advanced cervical cancer who achieved complete remission 10 weeks after drug infusion with only mild and controllable adverse effects (40).

The results of these studies indicate that TILs therapy has demonstrated effectiveness and safety in treating advanced cervical cancer in the US and Europe. However, its exploration in China remains nascent. Currently, Zhilin Biotech’s ZLT-001, Grit Biotechnology’s GT101 and GT201, and Jun Sai Biotech’s GC101 and GC203 have received clinical trial approval and are currently undergoing corresponding trials. If found effective, they could offer new treatment options for patients with metastatic or recurrent cervical cancer (**Table 1**).

**Table 1:** Summary of clinical studies of TILs therapy in cervical cancer (Available at https://clinicaltrials.gov, accessed on 1 November 2024).

No.	NCT number	Title	Phase and status	Regimen	Enrollment	Duration	Sponsor	Results (Refs.)
1	NCT05366478	A Clinical Study of LM103 Injection in the Treatment of Advanced Solid Tumors	Phase 1 (Recruiting)	TILs(LM103)	15	2022.05.30-2029.05.30	Suzhou BlueHorse Therapeutics Co., Ltd., China	–
2	NCT04674488	TILs for Treatment of Metastatic or Recurrent Cervical Cancer	Early Phase 1 (Recruiting)	TILs	15	2020.12.09-2024.11.09	Shanghai OriginCell Therapeutics Co., Ltd., China	–
3	NCT05393635	ITIL-168 in Advanced Solid Tumors (DELTA-2)	Phase 1 (Withdrawn)	ITIL-168	0	2022.08-2022.12.08	Instil Bio	–
4	NCT03108495	Study of LN-145, Autologous Tumor Infiltrating Lymphocytes in the Treatment of Patients with Cervical Carcinoma	Phase 2 (Active, not recruiting)	TILs (LN-145). TILs (LN-145) + Pembrolizumab (US Only)	189	2017.06.22-2030.12	Iovance Biotherapeutics, Inc., United States, France, Germany, Italy, Netherlands, Spain, Switzerland, United Kingdom	ORR(LN-145): 44.4% (12 of 27)-1 CR, 9 PR, 2 uPR, DCR was 89% at 3.5-month median study follow-up with 11/12 patients maintaining their response. Improved responses were observed in 4 patients with longer follow-up (62).
								ORR(LN-145 + Pembrolizumab): 50.0% (5 of 10)-1 CR, 4 PR (51).
5	NCT06191900	Clinical Study of GT201 in the Treatment of Advanced Gynecological Tumors (Advanced Cervical Cancer)	Not Applicable (Recruiting)	GT201 injection	26	2023.06.05-2026.06.05	Grit Biotechnology, China	–
6	NCT05475847	Study of C-TIL052A Cell Therapy in Advanced Cervical Cancer (TIL)	Phase 1 (Recruiting)	C-TIL052A injection followed by injection of IL-2	20	2022.07-2025.07	Fudan University, China	–
7	NCT04443296	Study of Tumor Infiltrating Lymphocytes Following CCRT in the Treatment of Patients With Cervical Carcinoma (TIL-Cx)	Phase 1 (Unknown status)	Cisplatin based concurrent chemoradiotherapy(CCRT) combined with tumor-infiltrating lymphocyte (TILs)	10	2019.10.12-2021.12.31	Sun Yat-sen University, China	ORR: 75.0% (9 CR of 12)-with a disease control duration of 9–22 months (63).
8	NCT01585428	Immunotherapy Using Tumor Infiltrating Lymphocytes for Patients With Metastatic Human Papillomavirus-Associated Cancers	Phase 2 (Completed)	Fludarabine + Cyclophosphamide + Young TILs + Aldesleukin	29	2012.04.13-2016.08.01	National Cancer Institute (NCI), United States	ORR: 33.3% (3 of 9)-2 CR (ongoing 22 and 15 months after treatment, respectively), 1 PR (3 months in duration) (60).
								ORR: 27.8% (5 of 18)-2 responses were complete (CR) and are ongoing 67 and 53 months after treatment; 3 responses were partial (PR) and of 3 months duration (15).
9	NCT05107739	A Study of DeTIL-0255 in Adults With Advanced Malignancies	Phase 1 (Terminated)	Drug Product De-TIL-0255	5	2021.12.22-2023.05.09	Nurix Therapeutics, Inc., United States	–
10	NCT06630611	Evaluation of a Pragmatic Approach to Adoptive Cell Therapy (ACT) Using an IL2 Analog (ANV419) *vs* High Dose IL2 After Tumor Infiltrating Lymphocytes (TIL) Therapy in Patients With Melanoma, NSCLC and Cervical Cancer (PragmaTIL) (PragmaTIL)	Phase 2 (Not yet recruiting)	TILs + High dose IL-2 *vs* TILs + IL-2 analog	40	2024.10-2029.09	Vall d’Hebron Institute of Oncology, Spain	–
11	NCT05342506	Clinical Study on the Safety, Pharmacokinetics, and Efficacy of ScTIL (Genetically Modified Tumor Infiltrating Lymphocytes) in the Treatment of Gynecological Malignancies	Phase 2 (Unknown status)	Toripalimab + ScTIL	30	2022.04.18-2024.05.31	Peking Union Medical College Hospital, China	–
12	NCT06241781	Autologous Tumor Infiltrating Lymphocytes (GT101 Injection) in Patients With Recurrent or Metastatic Cervical Cancer	Phase 2 (Recruiting)	GT101 injection *vs* Gemcitabine injection	64	2024.04.02-2027.01.31	Grit Biotechnology, China	–
13	NCT06626256	STIL101 for Injection for the Treatment of Locally Advanced, Metastatic or Unresectable Pancreatic Cancer, Colorectal Cancer, Renal Cell Cancer, Cervical Cancer and Melanoma	Phase 1 (Not yet recruiting)	Cyclophosphamide + Fludarabine + TILs(STIL101) + Aldesleukin	12	2024.12.30-2027.10.30	City of Hope Medical Center, United States	–
14	NCT04766320	Study on TIL for the Treatment of r/r Gynecologic Tumors	Phase 1 (Recruiting)	TILs injection	15	2021.01.04-2025.01.31	Shanghai 10th People’s Hospital, China	A patient treated with combination of TILs and PD1 achieved complete response 10 weeks after one-time TILs infusion (40).
								ORR: 40.0% (2 of 5)-1 CR (19.5 months in duration), 1 PR (16.5 months in duration), 3 SD (ongoing 6.3, 5.3 and 8.5 months after treatment) (140).
15	NCT06375187	Engineering Tumor Infiltrating Lymphocytes Injection (GC203 TIL) for the Treatment of Advanced Malignant Solid Tumors (KUNLUN-001)	Phase 1 (Recruiting)	GC203 TILs	18	2024.05.29-2027.05.01	Shanghai Juncell Therapeutics, China	–
16	NCT05902520	Adoptive Cell Therapy Using Cancer Specific CD8+ Tumor Infiltrating Lymphocytes in Adult Patients With Solid Tumors (ACT)	Phase 1 (Recruiting)	DP CD8 TILs (subpopulation of CD8 TIL highly enriched for tumor reactivity) + Low dose IL-2	18	2023.06.19-2026.05.19	AgonOx, Inc., United States	–
17	NCT05098171	Study on Signal Switch Receptor Modified TIL for the Treatment of Advanced Gynecologic Tumors	Early Phase 1 (Recruiting)	Signal Switch Receptor Modified TILs	50	2021.09.26-2025.09.25	Shanghai Juncell Therapeutics, China	–
18	NCT05468307	Study on TIL Engineered With Membrane-Binding Cytokine for the Treatment of Advanced Gynecologic Tumors	Early Phase 1 (Recruiting)	Membrane Bound Cytokine Modified TILs	50	2022.03.10-2025.03.10	Shanghai Juncell Therapeutics, China	–
19	NCT05724732	Exploratory Clinical Study of Autologous Tumor-infiltrating Lymphocyte Injection (GT201) for Advanced Gynecologic Tumors	Early Phase 1 (Recruiting)	TILs(GT201)	24	2023.02.01-2025.05.01	RenJi Hospital, China	–

TILs, Tumor-infiltrating lymphocytes; ACT, adoptive cell therapy; ORR, Objective Response Rate; DCR, Disease Control Rate; CR, Complete Response; PR, Partial Response; SD, Stable Disease; IL-2, interleukin-2; CCRT, concurrent chemoradiotherapy.

### 5.2 Genetically modified TILs

Genetically modified TILs (GM-TILs) refer to engineered TILs modified via gene-editing technologies—such as CRISPR/Cas9, TALEN, or viral vector transduction—to enhance their antitumor activity, persistence, or resistance to immunosuppressive signals within the tumor microenvironment (TME). This strategy aims to overcome limitations of conventional TIL therapy, including T cell exhaustion, restricted antigen recognition, and immunosuppressive TME influences.

Currently, GM-TIL applications in cervical cancer primarily focus on two strategies: *CRISPR/Cas9*-mediated knockout of immunosuppressive targets and cytokine signaling enhancement. The first approach is exemplified by GT316, which employs CRISPR-based dual knockout of undisclosed immunomodulatory targets (identified via genomic screening) to reduce T cell exhaustion and amplify antitumor efficacy. In a Phase I trial (NCT06145802), a heavily pretreated cervical cancer patient achieved a complete response (CR) lasting 32 weeks following infusion of 1.0×10^10 GT316 cells, with no dose-limiting toxicity observed. This response correlated with TIL expansion and elevated serum IFN-γ levels post-treatment, potentially linked to reduced TIL exhaustion phenotypes, though the precise targets require further validation (64). The second strategy, represented by GT201, involves engineering TILs to express membrane-bound IL-15 (mbIL-15), thereby enhancing persistence and reducing reliance on exogenous IL-2. In a Phase I trial (NCT05430360), a refractory cervical cancer patient infused with 5.3×10^9 GT201 cells achieved a partial response (PR) lasting 30 weeks, with detectable functional TILs persisting for one month post-infusion (65, 66). Both strategies demonstrated enhanced cytotoxicity in preclinical models (64, 65), and early clinical data suggest that targeting T cell exhaustion (e.g., GT316) or cytokine signaling (e.g., GT201) may overcome barriers such as IL-2 deficiency or T cell dysfunction in the cervical cancer TME (64, 66, 67). Additionally, alternative strategies—including PD-1 or TGFBR2 knockout—have shown promise in melanoma or ovarian cancer models (67–69), though data in cervical cancer remain unreported. Their applicability may depend on cervical cancer-specific TME features, such as elevated TGF-β levels (68, 69).

Despite preliminary validation of GM-TIL safety in early trials, significant limitations persist: small sample sizes (single-case reports), unclear mechanisms of genetic modifications (64, 65), and tumor heterogeneity potentially compromising therapeutic generalizability. Future directions should prioritize HPV-specific targeting (e.g., E6/E7 TCR-T), multiplex gene editing (e.g., PD-1 KO combined with IL-15 expression), and optimization of TIL expansion protocols to minimize exhaustion phenotypes (70, 71). Rigorous validation through cervical cancer-specific preclinical models and randomized trials will be critical to establish efficacy (72, 73).

### 5.3 Combination therapy with TILs

For patients with recurrent or metastatic cervical cancer, traditional treatment options are limited. Although TILs therapy has advantages, it may not be effective for all patients. A combination of different treatment can synergistically enhance the efficacy of cancer therapy.

#### 5.3.1 TILs in combination with chemotherapy

Chemotherapy can enhance immune responses, and combining treatments can increase efficacy. In a clinical study, Ramos et al (74) showed that the combination of immunotherapy and chemotherapy can overcome immunosuppression, reduce the accumulation of immunosuppressive cells, enhance the frequencies of antigen-presenting cell/CD8+ T cells, and boost anti-tumor immunity. Several studies indicate that the immunogenicity of drug-resistant tumor cells and the host’s immune response are key factors affecting chemotherapy efficacy (75). The combination of chemotherapy and TILs therapy has shown improved clinical outcomes in the treatment of head and neck cancers and ovarian cancer (76, 77). These findings indicate that combining chemotherapy with TILs therapy holds promising potential for improving cancer patient prognosis.

#### 5.3.2 TILs in combination with radiotherapy

Radiotherapy (RT) was believed to inhibit the immune function of the body because of its lethality to lymphocytes. However, the view of RT has dramatically changed in recent years, and it is now widely accepted that RT can provoke a systemic immune response. Immunotherapy combined with radiotherapy can produce not only local tumor control but also a systemic effect on remote and non-irradiated tumor deposits. RT increases the expression of MHC-I in tumor cells (78), which facilitates the recognition of tumors by CD8+ T cells and reduces immune escape (79). Additionally, radiation destroys tumors, releasing antigens and pro-inflammatory cytokines (e.g., CXCL16) that recruit immune cells (80, 81). Immune enhancement is believed to occur at the irradiated tumor site with increased T-cell proliferation (82). Research has shown that the abundance of TILs in tumor samples collected before the start of concurrent CCRT is a prognostic factor for head and neck cancer (83) and colorectal cancer (84). Huang et al. (63) demonstrated that TILs treatment combined with concurrent chemoradiotherapy is safe and effective for patients with locally advanced cervical cancer.

#### 5.3.3 TILs in combination with immune checkpoint inhibitors

In the cervical cancer immune microenvironment, hyperactivation of the PD-1/PD-L1 pathway promotes immune evasion by suppressing T cell functionality, while blockade of this pathway reverses TIL exhaustion and enhances antitumor activity (85–87). Studies demonstrate that HPV infection upregulates PD-L1 expression on both tumor cells and TILs, correlating with poor prognosis; however, PD-1/PD-L1 inhibitors restore TIL function by interrupting immunosuppressive signaling (88–90). Clinical evidence confirms that combining TILs with immune checkpoint inhibitors (ICIs, e.g., anti-PD-1 monoclonal antibodies) significantly improves outcomes: in metastatic cervical cancer patients, combination therapy achieves ORR of 25%-50%, with median progression-free survival (PFS) and overall survival (OS) reaching 6.1 months and 11.3 months, respectively (62, 91). Iovance’s clinical trial evaluating TILs (e.g., LN-145) combined with pembrolizumab reported an ORR of 50%, surpassing monotherapy outcomes (ORR 44.4%) (50, 51, 62). Mechanistically, ICIs synergize with TILs by alleviating PD-1/PD-L1-mediated T cell suppression and amplifying tumor antigen-specific cytotoxicity, thereby remodeling the immunosuppressive microenvironment (20, 88). Despite the promise of combination strategies, challenges persist in optimizing patient selection criteria (e.g., PD-L1 expression, HPV status) and elucidating resistance mechanisms. Future multicenter randomized trials are imperative to validate long-term safety and survival benefits (51, 91).

#### 5.3.4 TILs in combination with targeted therapy

Anti-angiogenic drugs are a conventional treatment for advanced cervical cancer. These drugs remodel and normalize tumor vasculature, thereby reducing hypoxia and allowing effector T cells to infiltrate effectively. However, no clinical studies have yet reported the outcomes of combination of TILs and anti-angiogenic drugs. In a study combining ACT with targeted therapy, Morisaki et al. (92) cultured cytokine-activated killer cells with cetuximab. They found that the combination significantly enhanced cytotoxicity but this enhancement was inhibited by the addition of excess human immunoglobulin, suggesting that antibody-dependent cytotoxicity was involved in this mechanism. The findings of these *in vitro* studies and from animal models demonstrate that anti-tumor drugs can directly or indirectly influence the function of TILs, which may benefit the clinical immunotherapy of cervical cancer and serve as a theoretical basis for future clinical translation research.

#### 5.3.5 Other combinatorial therapeutic strategies

Although cervical cancer tissues exhibit high mutation burden and immunogenicity, the TME is highly immunosuppressive. Regardless of the degree of tumor-specific or neoantigen-specific TILs, reinfused TILs are still affected by TME-mediated mechanisms, such as IL-10, TGF-β, nitrogen metabolism products, low pH, high lactate, and hypoxia (93), a challenge faced by any T cell migrating to the TME.

The immune microenvironment of cervical cancer contains a large number of suppressive immune cells, including Treg cells, MDSCs, tumor-associated macrophages (TAMs), and tumor-associated neutrophils (TANs) (94). These factors not only impair the homing of T lymphocytes but also reduce their activity, making it difficult to sustain persistent anti-tumor immune effects. Furthermore, MDSCs not only possess immunosuppressive function but also promote tumor angiogenesis, thereby aiding metastasis and helping tumors evade immune surveillance (95). Although lymphodepletion before TILs infusion can eliminate some suppressive immune cells, it is associated with significant side effects.

To overcome the immunosuppressive constraints of the tumor microenvironment (TME), current strategies focus on converting inhibitory signals into stimulatory cues to counteract T cell suppression. Key approaches include: (1) Epigenetic modulation using DNA methyltransferase inhibitors (DNMTi) to downregulate Treg- and MDSC-associated inhibitory factors (e.g., FOXP3, ARG1), thereby enhancing CD8+ T cell cytotoxicity and supporting CD4+ T cell function (96–98); (2) Targeting TAMs by blocking CD47/SIRPα or TREM2 signaling to reduce M2 macrophage recruitment (99, 100), while JMJD1C inhibitor 193D7 suppresses IFNγ signaling to diminish Treg infiltration and synergize with TILs (101); (3) Bifunctional antibodies such as M7824 and SHR-1701 (targeting PD-L1/TGF-β), which simultaneously block checkpoint inhibition and neutralize TGF-β-mediated T cell dysfunction, with preclinical studies demonstrating enhanced TILs activity (102–104) and (4) *In situ* TILs activation via intratumoral delivery of mRNA encoding membrane-anchored anti-CD3 single-chain variable fragments (scFv) using lipid nanoparticles (LNPs), which directly engineer TAMs and tumor cells to activate and expand TILs, achieving robust antitumor efficacy in melanoma (B16F10) and colon cancer (MC38) models and synergistically reversing PD-1 resistance when combined with anti-PD-1 therapy (105). This *in situ* approach bypasses complex ex vivo TIL expansion, locally remodels the TME to amplify T cell cytotoxicity, and offers a novel therapeutic avenue for cervical cancer. Future efforts must optimize the dosing schedules and the sequence of combination therapies while advancing TME subtype-guided precision interventions.

### 5.4 Prognostic biomarkers for cervical cancer TILs therapy

Current studies have identified critical prognostic biomarkers associated with cervical cancer TILs therapy. CD8+ TILs density serves as a pivotal indicator, with higher infiltration levels significantly correlating with prolonged recurrence-free survival (RFS) and overall survival (OS) (106). Conversely, FasL overexpression in tumor cells negatively correlates with CD45+ TILs infiltration (OR=9, p=0.01), suggesting FasL-mediated TILs apoptosis may drive immune evasion (106). PD-L1 expression (positive in 68% of cases) and its Combined Positive Score (CPS) exhibit dual implications: PD-L1 positivity may predict responsiveness to immune checkpoint inhibitors, yet its co-occurrence with TILs exhaustion phenotypes underscores the need for combinatorial strategies (107). Epigenetic markers such as ERBB3 promoter methylation reduce TILs infiltration by suppressing immune-related RNA pathways, offering novel avenues for patient stratification (108). These biomarkers guide both prognosis and therapeutic optimization: high CD8+ TILs density and low ERBB3 methylation may identify candidates for adoptive TILs therapy, while FasL inhibitors could counteract immunosuppressive microenvironments (106, 108). However, challenges persist due to TILs functional heterogeneity (e.g., dynamic equilibrium between exhausted and activated subsets) and limited multicenter validation (107). Future integration of spatial transcriptomics and standardized AI-driven quantification is essential to enhance prognostic precision and advance personalized therapies (109).

### 5.5 Potential advantages of cervical cancer TILs therapy

Compared to other solid tumors (e.g., colorectal cancer, glioblastoma), cervical cancer TILs therapy demonstrates unique advantages driven by HPV-associated antigen immunogenicity and microenvironmental plasticity. Persistent HPV infection induces stable expression of E6/E7 viral antigens, providing TILs with highly specific targets to overcome tumor heterogeneity through polyclonal T cell responses (110–113). Preclinical studies reveal that HPV antigen-specific TILs combined with anti-PD-1 therapy significantly enhance CD8+ T cell infiltration while reducing immunosuppressive cells (e.g., Tregs, MDSCs), achieving higher tumor regression rates than breast or lung cancer models (111, 113). In contrast, TILs in other solid tumors (e.g., MC38 colorectal cancer) are prone to exhaustion due to antigen heterogeneity and immunosuppressive pathways (e.g., KLF5/COX2, MIF/CD74), necessitating genetic editing (e.g., Cbl-b knockout) or metabolic reprogramming (e.g., PGC-1α overexpression) to restore functionality (114–116). Additional advantages of cervical cancer TILs include: (1) viral origin of HPV antigens enhancing antigen presentation efficiency (110, 117); (2) synergy with mRNA vaccines to convert immunologically ‘cold’ tumors (110); and (3) activation of non-classical immune subsets (e.g., γδ T cells) for coordinated antitumor activity (117). Future priorities include HPV multi-epitope TIL therapies combined with metabolic modulation, though efficacy in HPV-negative cervical cancer remains unresolved (115).

## 6 TILs treatment-related adverse events

TILs therapy generally has mild side effects and a relatively good safety profile. Tevanovic et al. (15) reported that patients with metastatic cervical cancer who had previously received platinum-based chemotherapy or chemoradiotherapy experienced no acute infusion-related toxicities or autoimmune adverse events following TILs treatment. The AEs associated with TILs therapy are mainly linked to the NMA chemotherapy conditioning regimen and IL-2 treatment (118). Almost all patients who receive the NMA regime experience bone marrow suppression, including neutropenia, lymphopenia, anemia, and thrombocytopenia (119, 120). These side effects can be managed with granulocyte-colony stimulating factor (G-CSF) or blood product transfusions (119, 121, 122). Non-hematologic toxicities related to NMA regimens include fever, diarrhea, edema, hyperbilirubinemia, and fludarabine-induced neurotoxicity (123, 124). Some patients may develop secondary pulmonary infections or reactivation of herpes zoster, which can be managed with standard post-chemotherapy prophylactic treatments (125). Toxicities directly related to the infusion of TILs product are extremely rare and are often difficult to distinguish from reactions caused by residual IL-2 in the TILs product (126, 127). Common allergic reactions include fever, pruritus and dyspnea (128).

The infused TILs are directly targeted toward and attack neoantigen-expressing tumor cells. However, many tumor antigens that are therapeutic targets are also expressed in normal tissues. Therefore, TILs may recognize antigens in normal tissues and cause autoimmune reactions in healthy tissues. Reports have indicated that adoptive T cells can target not only the tumor cells with specific antigens but also normal skin cells and uveal cells, leading to conditions such as vitiligo or uveitis, with an occurrence rate of approximately 35% and 15%, respectively. These side effects can be mitigated with the topical application of corticosteroids (125, 128). Moreover, high doses of IL-2 can cause several serious, dose-dependent systemic adverse events. One of the most significant AEs is capillary leak syndrome, the complex mechanism of which includes IL-2-mediated promotion of the synthesis and release of inflammatory mediators and an increase in vascular permeability, causing extravasation of fluids and proteins, resulting in interstitial edema and, in severe cases, multi-organ damage (129). IL-2 also stimulates the release of inflammatory cytokines, which can lead to cytokine storm, characterized by high fever, diarrhea, nausea, vomiting, anemia, thrombocytopenia, and elevated transaminase levels (130).

Several organs, including the heart, lung, kidney, and central nervous system, can be affected by IL-2 toxicity, but these effects are manageable with standard clinical care. Preparative lymphodepleting chemotherapy has been reported to reduce IL-2-related AEs, as lymphocytes in immune-active individuals are the primary source of cytokines responsible for IL-2-related side effects (131). Engineering IL-2 prodrugs (ProIL-2) have also been shown to significantly reduce IL-2 toxicity and mortality without compromising the antitumor efficacy of TILs therapy (132). In summary, although there are toxicities associated with TILs therapy, most side effects are low-grade and can be managed with standard clinical treatments.

## 7 Challenges and future prospects of TILs therapy

Tumor-infiltrating lymphocytes (TILs), as autologous immune cells derived from patients, exhibit unique advantages including high specificity, multi-target recognition capability, and low off-target toxicity. The proportion of tumor-specific T cells in TILs is significantly higher than in other adoptive cell therapies (ACTs), with approximately 60% of TILs capable of recognizing tumor neoantigens through diverse T cell receptor (TCR) repertoires targeting mutated tumor epitopes, thereby overcoming tumor heterogeneity and suppressing immune evasion (133). This polyclonal nature enables TILs to simultaneously target multiple tumor-associated antigens (TAAs), avoiding therapeutic failure due to single-antigen loss (134). Furthermore, TILs inherently express chemokine receptors (e.g., CCR5, CXCR3), facilitating homing to tumor sites and enhanced infiltration into the tumor microenvironment (TME) post-reinfusion, which amplifies their localized cytotoxic efficacy (135). Compared to genetically engineered T cell therapies (e.g., CAR-T), TILs achieve broad coverage of unknown neoantigens without requiring ex vivo engineering, rendering them particularly promising for solid tumor treatment (135).

Despite its potential, TILs therapy faces several limitations. The initial step—surgical resection or biopsy to obtain tumor tissue—is invasive and poses challenges for tumors in anatomically complex or high-risk locations, with potential risks of inadequate sampling or procedural complications. Following isolation, TILs expansion requires specialized equipment and technical expertise. Moreover, the unpredictable yield of TILs from tumor specimens may result in insufficient cell numbers for ex vivo culture, compounded by the lack of standardized protocols across laboratories, leading to variability in therapeutic efficacy. Additionally, the prolonged manufacturing timeline—currently optimized to 22 days by Iovance (41) but typically requiring 6–8 weeks in other protocols—may delay treatment for rapidly progressing tumors, necessitating bridging therapies (e.g., localized radiotherapy or systemic chemotherapy) during this window, as exemplified by clinical trial GT-CD-CHN-101-02. Furthermore, the personalized nature of TILs therapy precludes batch production, requiring costly Good Manufacturing Practice (GMP)-compliant facilities, trained personnel, and specialized instrumentation, which exacerbates healthcare resource inequities.

It is important to note that TILs are a group of heterogeneous lymphocytes in the tumor stroma with different specificities. Although TILs are abundant with tumor-specific T cells compared with circulating T cells, they also contain non-specific cells. Therefore, not all T cells within TILs products have potent tumor reactivity. For instance, tumors with low levels of unique antigens are likely to contain low levels of tumor-specific TILs, which may result in poor therapeutic efficacy. A critical challenge arises from tumor antigen-specific T cells becoming hyporesponsive owing to continuous encounters with tumor antigens. This phenomenon is accompanied by the upregulation of numerous inhibitory receptors, causing T-cell exhaustion and a state of hyporesponsiveness (20). Fortunately, current imaging and single-cell analysis technologies enable us to precisely reveal cell and molecular interactions in animal models and patients, providing an in-depth description of the precise anatomical localization of immune activation and effector functions. These technologies can also identify mechanisms that lead to either successful or suppressed anti-tumor immune responses. Consequently, identifying and selectively expanding pre-defined tumor-specific T cells targeting unique tumor antigens may help avoid TILs hyporesponsiveness.

Another major limitation lies in the prolonged survival of TILs *in vivo*, which is a crucial factor for complete response duration and prevention of recurrence. However, the infused TILs have a brief survival duration *in vivo*, and after the number of TILs cells decreases, tumor cells are prone to escape from immune surveillance. Notably, in other adoptive cell therapy (ACT) approaches, the brief survival duration of the infused specific T cells explains the low therapeutic efficacy in non-responding patients (136). TILs therapy faces a similar challenge. To address this, pretreating TILs with cytokines before reinfusion increases the survival and function of T cells *in vivo* (137). Nevertheless, long-term use of these cytokines is limited by the associated severe side effects. Recent studies (138) have shown that recombinant human IL-2-loaded nanoparticles can controlly release IL-2 to tumors, thus reducing systemic side effects by minimizing systemic IL-2 dosage.

Alternatively, insights from other adoptive immunotherapies suggest that genetically modifying T cells could enhance their persistence. For example, artificial T cell adapter molecules (ATAMs) have been shown to improve the persistence of TCR-gene-transduced T cells. ATAMs are gene-modified CD3ζ with the intracellular domain of 4-1BB inserted in the middle of CD3ζ (139). Additionally, TILs often contain large numbers of effector memory T cells, which express chemokine receptors after being stimulated by tumor antigens *in vivo* (135). Therefore, during preparation, selecting a certain number of memory T cells for expansion and reinfusion can enhance immune surveillance, thereby improving the persistence of the therapy.

To improve the efficacy of TILs-based immunotherapy in solid tumors, researchers are continuously exploring different combination strategies. Beyond traditional therapies (e.g., chemotherapy, radiation), interdisciplinary innovations such as lipid nanoparticle (LNP)-mediated delivery systems hold significant potential. Although preclinical studies have demonstrated strong anti-tumor activity with several combinations, only combinations with ICIs have been clinically validated. One significant challenge is the difficulty in selecting an optimal preclinical model to assess the anti-tumor activity of combination therapies. Another significant challenge is that using combination therapies may increase the risk of immune-related adverse events (IMAEs) and medical costs, as selecting inappropriate combination therapies can expose patients to significantly higher toxicity. Optimizing the treatment regimen, including dose, timing, and sequence, presents another challenge in the development of combination therapies. Finally, determining the appropriate combination therapy and identifying biomarkers that predict treatment response require large-scale clinical trials. Despite these challenges, we envision a future in which personalized combination therapies are designed based not only on the individual patient’s immune microenvironment but also on other predictive biomarkers. A comprehensive evaluation system integrating genomics, transcriptomics, immune analysis, and the microbiome will be essential to select patients most likely to benefit from combination therapies.

## 8 Conclusion

The treatment of recurrent or metastatic cervical cancer is a prolonged and complex process, making the pursuit of innovative and safe therapeutic approaches a primary focus of clinical research. Tumor-infiltrating lymphocyte (TILs) therapy, characterized by its strong tumor-targeting specificity, minimal off-tumor toxicity, and favorable safety profile, has emerged as a novel precision-based personalized treatment modality. Clinical studies in advanced cervical cancer have demonstrated its significant therapeutic efficacy. However, the immunosuppressive tumor microenvironment (TME) remains a major challenge for TILs therapy. Combination strategies, such as TILs with PD-1/PD-L1 inhibitors, have shown promising results in early trials. Additionally, reducing reliance on high-dose IL-2 infusion and lymphodepletion has improved safety while maintaining clinical benefits in some studies. Genetically modified TILs hold potential to overcome current limitations and further enhance therapeutic outcomes. Despite these advantages, the labor-intensive and costly nature of TILs therapy, coupled with the complexity of tissue collection and manufacturing processes, has restricted its development to leading research institutions and companies in only a few countries. Nevertheless, TILs therapy retains immense potential for refinement and broader application, including the establishment of standardized and stable manufacturing protocols, enrichment of tumor-specific TILs populations, optimization of combination regimens, and identification of prognostic biomarkers. As a cutting-edge research area, TILs therapy offers renewed hope for improving survival rates and quality of life in patients with recurrent or metastatic cervical cancer.
